# Decoding RAGE in diabetes: a pathogenic signaling hub modulated by decoy receptors in diabetic complications

**DOI:** 10.3389/fcdhc.2026.1854214

**Published:** 2026-08-26

**Authors:** Yupa Srithongchai, Ai Harashima, Seiichi Munesue, Kumi Kimura, Yu Oshima, Mariko Tanaka, Hiroya Takahashi, Pathitta Sakulsak, Chisato Uramoto, Nontaphat Leerach, Yasuhiko Yamamoto

**Affiliations:** 1 Department of Biochemistry and Molecular Vascular Biology, Kanazawa University Graduate School of Medical Sciences, Kanazawa, Japan; 2 Princess Srisavangavadhana Faculty of Medicine, Chulabhorn Royal Academy, Bangkok, Thailand

**Keywords:** advanced glycation end-products (AGEs), diabetic complications, inflammation, NF-κB, receptor for AGEs (RAGE)

## Abstract

Diabetes is characterized by chronic hyperglycemia and dyslipidemia, which impose sustained metabolic stress and drive the development of diabetic complications. In this review, we position the axis between advanced glycation end-products (AGEs) and the receptor for AGEs (RAGE) as a central hub that couples metabolic stress to persistent cellular stress and inflammatory responses. Ligand engagement of full-length membrane-bound RAGE (mRAGE) enhances reactive oxygen species (ROS) generation and activates nuclear factor kappa B (NF-κB), establishing a self-amplifying feed-forward loop that promotes RAGE expression, inflammation and further AGE accumulation. RAGE exhibits marked structural heterogeneity: in addition to signaling-competent mRAGE, soluble decoy receptors—including soluble RAGE (sRAGE) produced by ectodomain shedding and endogenous secretory RAGE (esRAGE) generated by alternative splicing—sequester ligands and attenuate downstream signaling. Moreover, RAGE recognizes a broad ligand repertoire beyond AGEs, such as S100 proteins and high-mobility group box 1 (HMGB1), reinforcing its role as an integrator of metabolic and sterile inflammatory cues. Notably, RAGE also mediates physiological transport of oxytocin across the blood–brain barrier (BBB), a process implicated in oxytocin-dependent social behaviors, without activating canonical, pro-inflammatory RAGE signaling. Accordingly, therapeutic strategies should selectively suppress pathogenic, ligand-driven RAGE signaling and/or augment decoy RAGE activity while preserving RAGE-dependent oxytocin transport.

## 1 Introduction

**Figure 1:**
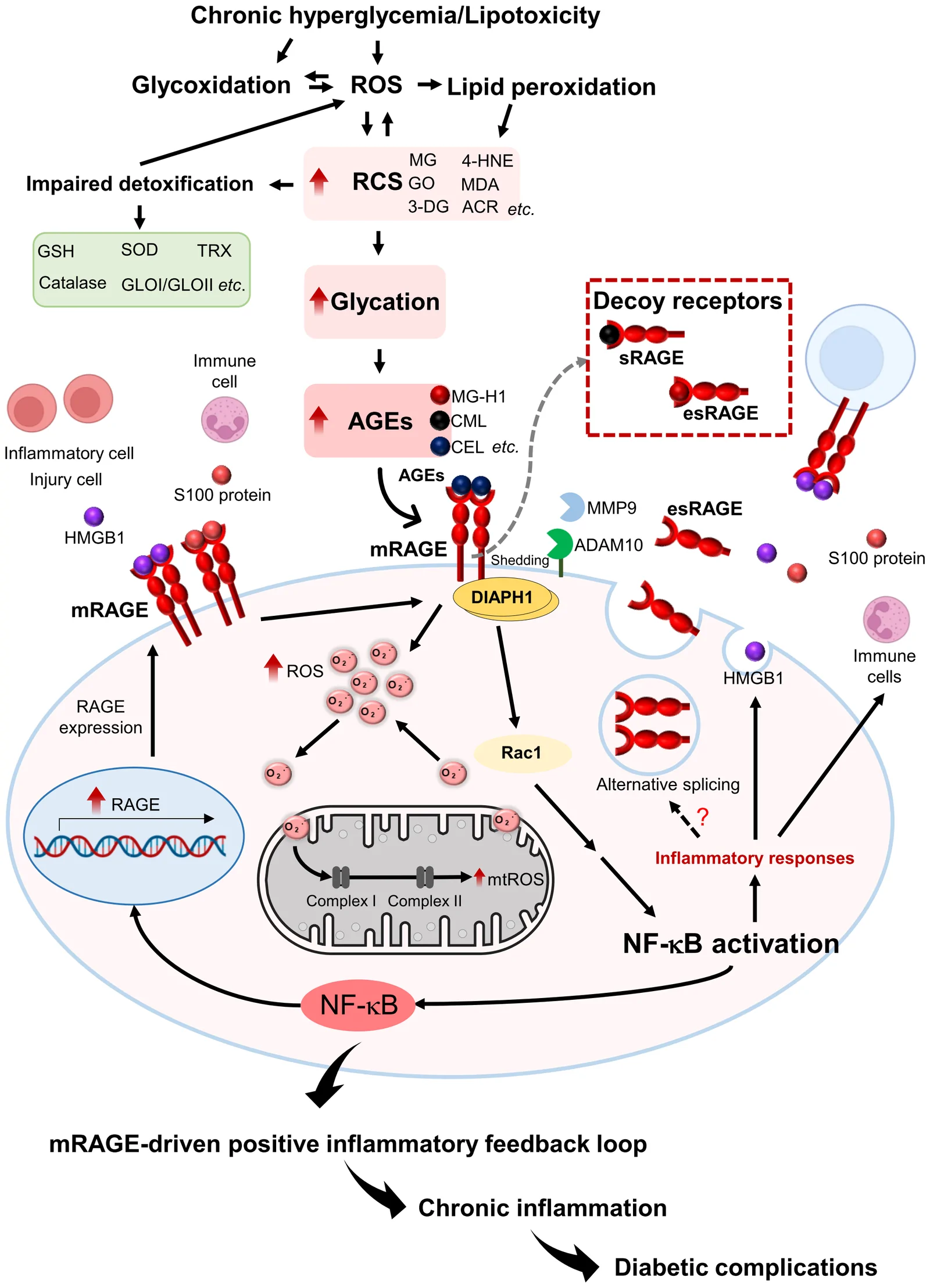
Pathogenic AGEs–mRAGE signaling axis in diabetic complications. Chronic hyperglycemia and lipotoxicity induce metabolic overload in diabetic tissues. These conditions promote glycolytic stress, glycoxidation, mitochondrial dysfunction, and reactive oxygen species (ROS) generation. Increased ROS further drives lipid peroxidation and glycoxidation, leading to the accumulation of reactive carbonyl species (RCS) such as methylglyoxal (MG), glyoxal (GO), 3-deoxyglucosone (3-DG), 4-Hydroxynonenal (4-HNE), Malondialdehyde (MDA), and acrolein (ACR). These processes enhance the formation of advanced glycation end-products (AGEs), including Nε-carboxymethyl-lysine (CML), Nε-carboxyethyl-lysine (CEL), and methylglyoxal-derived hydroimidazolone (MG-H1). AGEs, together with other RAGE ligands such as high-mobility group box 1 (HMGB1) and S100 proteins, bind to membrane-bound full-length RAGE (mRAGE). mRAGE is the signaling-competent receptor isoform. Ligand binding and receptor clustering activate intracellular pathways involving diaphanous-related formin 1 (DIAPH1), small GTPases, Rac1, and ROS formation. These pathways lead to nuclear factor kappa B (NF-κB) activation and the induction of pro-inflammatory cytokines, chemokines, adhesion molecules, and RAGE itself. Upregulation of mRAGE further amplifies ligand-induced signaling. This creates a positive-feedback loop that sustains redox imbalance and chronic inflammation. These events may contribute to tissue injury and the onset and progression of diabetic complications. Soluble RAGE isoforms, including soluble RAGE (sRAGE) and endogenous secretory RAGE (esRAGE), are shown as decoy receptors. They buffer extracellular ligand availability and may attenuate the magnitude and persistence of mRAGE signaling.

Diabetes mellitus (DM) is increasingly recognized as a systemic metabolic disorder that extends beyond impaired glucose homeostasis and involves chronic metabolic stress, nutrient imbalance, and cellular dysfunction (1). Under conditions of sustained hyperglycemia and lipotoxicity, dysregulated glycolysis and oxidative stress promote the generation of reactive carbonyl species (RCS), including methylglyoxal and other dicarbonyl intermediates (2–4). When endogenous detoxification capacity is overwhelmed, these reactive intermediates drive nonenzymatic glycation, leading to the progressive formation and accumulation of advanced glycation end-products (AGEs) (4). Glycoxidation is known to act as a biochemical bridge between glycation and oxidative stress. Under diabetic conditions, classical early glycation reactions are accelerated, leading to the formation of Schiff bases and Amadori products, with HbA1c serving as a representative example. These intermediates can undergo reactive oxygen species (ROS)-mediated oxidative degradation, resulting in increased production of RCS. The resulting RCS contribute to AGE formation and can further amplify oxidative stress through protein modification, mitochondrial dysfunction, and receptor-mediated inflammatory reactions. Thus, glycoxidation provides a mechanistic link between hyperglycemia, carbonyl stress, and sustained ROS production (**Figure 1**).

The receptor for advanced glycation end-products (RAGE) is a pattern-recognition receptor that comprises multiple isoforms with distinct functional properties. Whereas soluble RAGE isoforms primarily act as ligand-sequestering decoy receptors, membrane-bound full-length RAGE (mRAGE) serves as the signaling receptor that transduces ligand engagement into intracellular pathways involving reactive oxygen species (ROS), stress-responsive kinases, and inflammatory transcriptional programs (5–8). A key mechanistic feature of this signaling process is the interaction of the cytoplasmic domain of mRAGE with diaphanous-related formin 1 (DIAPH1), which couples extracellular ligand recognition to intracellular signal propagation (9). These pathways converge on nuclear factor kappa B (NF-κB), thereby driving transcriptional programs that induce pro-inflammatory cytokines, chemokines, and adhesion molecules (9, 10). NF-κB-dependent upregulation of RAGE sustains receptor abundance at the cell surface, while inflammatory stress further enhances the generation of reactive dicarbonyl intermediates and AGEs, as well as the release of additional ligands, including high-mobility group box 1 (HMGB1) and S100 proteins (11, 12). Collectively, these interdependent processes establish a chronic feed-forward loop in which oxidative stress, glycoxidation, inflammation, and ligand accumulation perpetuate one another (**Figure 1**).

The magnitude and persistence of this signaling are further shaped by the balance between mRAGE and its soluble decoy isoforms, soluble RAGE (sRAGE) and endogenous secretory RAGE (esRAGE), which lack the transmembrane and cytoplasmic domains required for signal transduction and instead act by sequestering extracellular ligands (13–15). Within this framework, the AGEs–RAGE axis emerges as a central metabolic–inflammatory hub linking carbonyl stress, redox disequilibrium, innate immune activation, and progressive organ dysfunction (8). In this sense, RAGE is better understood not as an isolated receptor pathway, but as a systems-level integrator of metabolic danger signals that translates biochemical overload into sustained inflammatory remodeling across tissues (**Figure 1**).

Accordingly, this Review aims to advance current understanding of RAGE as a central pathogenic signaling hub in diabetes while providing a conceptual and mechanistic framework for the development of therapeutic strategies against diabetic complications. Building on this systems-level view, we propose a broader perspective of RAGE biology that incorporates two emerging concepts: ligand-induced oligomerization of mRAGE as a prerequisite for its intracellular signaling, and RAGE-mediated oxytocin transport across the blood–brain barrier (BBB) as a physiological function. These perspectives highlight the dual nature of RAGE as both a disease-promoting signaling receptor and a molecule with physiological functions, underscoring the need for therapeutic approaches that selectively suppress pathological RAGE signaling while preserving beneficial RAGE-dependent processes.

## 2 Metabolic stress, oxidative stress, carbonyl stress, and AGEs heterogeneity

Although the AGEs–RAGE axis serves as a major amplifier of redox-inflammatory signaling in diabetes, its chronic activation originates upstream in persistent metabolic overload. Type 2 diabetes (T2D) is characterized by sustained hyperglycemia and dyslipidemia, which impose prolonged bioenergetic and oxidative stress across insulin-sensitive, vascular, and parenchymal tissues (1, 10, 16). Excess glucose flux accelerates glycolytic throughput and, once mitochondrial oxidative capacity is exceeded, redirects intermediates into subsidiary pathways, including the polyol pathway, the hexosamine pathway, and diacylglycerol–protein kinase C signaling, while simultaneously enhancing MG formation as a reactive glycolytic byproduct (10, 16–19). The polyol pathway may also contribute to glycation, oxidative stress, and carbonyl stress under hyperglycemic conditions. Excess intracellular glucose is reduced to sorbitol by aldose reductase, with concomitant consumption of NADPH, and is subsequently converted to fructose by sorbitol dehydrogenase, generating NADH (10). Depletion of NADPH can impair glutathione-dependent antioxidant defense, whereas an increased NADH/NAD^+^ ratio may promote mitochondrial ROS production. In addition, fructose and its downstream metabolites can enhance non-enzymatic glycation and generate RCS, such as MG and 3-deoxyglucosone. In parallel, elevated free fatty acids promote mitochondrial dysfunction, incomplete β-oxidation, endoplasmic reticulum stress, and increased ROS generation (16–18). Together, these disturbances erode redox homeostasis and metabolic flexibility, thereby creating conditions that favor carbonyl stress.

As a result, RCS accumulate, with glycolytic stress driving the generation of reactive dicarbonyls such as MG, while lipid peroxidation produces additional carbonyl intermediates, including glyoxal and 3-deoxyglucosone (3, 4, 18). These highly reactive species modify proteins, lipids, and nucleic acids far more rapidly than glucose itself, thereby accelerating AGE formation. Under physiological conditions, the glyoxalase system, particularly glyoxalase 1 (GLO1), constrains MG accumulation (2, 3); however, in diabetes, excessive substrate burden and oxidative stress impair this detoxification pathway, resulting in sustained carbonyl stress (4, 18).

AGEs generated under these conditions comprise a structurally diverse group of adducts and crosslinks produced through nonenzymatic reactions involving reducing sugars, reactive dicarbonyls, lipid-derived intermediates, and oxidative processes (4, 18). In diabetes, major AGE species include *N*^ϵ^-carboxymethyl-lysine (CML), *N*^ϵ^-carboxyethyl-lysine (CEL), methylglyoxal-derived hydroimidazolone (MG-H1), pyrraline, and argpyrimidine, whereas crosslinking AGEs such as pentosidine, glucosepane, and methylglyoxal-lysine dimer (MOLD) accumulate in extracellular matrix proteins, promoting tissue stiffening and rendering these structures more resistant to proteolytic turnover (2–4, 18). Importantly, the relative abundance of these species is determined not only by glycemia, but also by tissue context, redox state, substrate availability, and glyoxalase activity (2, 3). Thus, the diabetic AGEs profile is determined not simply by hyperglycemia or the rate of AGE formation alone, but by the integrated balance among carbonyl flux, the efficiency of precursor detoxification pathways such as glyoxalase 1 (GLO1), and the half-life and turnover of AGE-modified biomacromolecules.

However, it remains unclear which individual AGE species, if any, serves as the principal pathogenic driver of diabetic complications *in vivo*, or whether tissue injury is more accurately explained by the cumulative and context-dependent actions of multiple AGE species. The biological significance of AGE accumulation is therefore determined not only by the chemistry of glycation itself, but also by how these modified ligands are sensed and translated into cellular responses (**Figure 1**).

## 3 RAGE as a multiligand pattern-recognition receptor: isoform balance and downstream signaling

RAGE has emerged as a central molecular integrator that links carbonyl stress and ligand accumulation to sustained intracellular signaling (7–9, 19). More broadly, RAGE is a multiligand pattern-recognition receptor that recognizes not only AGEs, but also a wide spectrum of endogenous danger-associated molecular patterns (DAMPs), including HMGB1, multiple S100 family proteins, and amyloid-β, and can also participate in the recognition or cellular handling of pathogen-associated molecular patterns (PAMPs), including lipopolysaccharide (LPS) and extracellular nucleic acids (7, 19, 20). This broad ligand repertoire places RAGE at the interface of metabolic stress, inflammation, and innate immune activation, allowing it to function as a molecular sensor that integrates diverse danger signals into common downstream responses. In immune cells, particularly macrophages, RAGE activation has been shown to promote NADPH oxidase-dependent ROS generation and pro-inflammatory signaling. Although direct evidence that RAGE activation itself increases RCS is limited, RAGE-induced ROS may indirectly enhance RCS formation through lipid peroxidation and glycoxidation. In addition, inflammatory activation of macrophages and microglia has been reported to increase MG production, suggesting that immune-cell activation can contribute to carbonyl stress (21). Thus, RAGE signaling may indirectly amplify RCS accumulation by promoting oxidative stress and inflammatory metabolic reprogramming, thereby establishing a feed-forward loop among AGEs -RAGE signaling, ROS production, and carbonyl stress.

The functional output of RAGE is determined not only by ligand availability, but also by the balance among distinct receptor isoforms with different biological properties (13–15). Among these isoforms, membrane-bound full-length RAGE (mRAGE) is expressed in a range of cell types implicated in diabetic complications, but is particularly abundant in immune cells, especially inflammatory cells such as neutrophils and macrophages/monocytes (20). mRAGE is the signaling-competent receptor form. It is a transmembrane member of the immunoglobulin superfamily composed of an extracellular V-type ligand-binding domain, two C-type domains, a transmembrane segment, and a short cytoplasmic tail required for intracellular signal transduction (5–7, 19). Although the cytoplasmic tail lacks intrinsic catalytic activity, it is indispensable for downstream signaling because it engages intracellular binding partners, most notably DIAPH1 (9). The identification of DIAPH1 as a binding partner for the cytoplasmic domain of mRAGE represented a key mechanistic advance in understanding how extracellular ligand recognition is coupled to intracellular signaling.

mRAGE oligomerization is required for efficient signal transduction, rather than being merely a passive consequence of ligand binding. mRAGE forms homodimers at the cell surface, and disruption of this self-association markedly suppresses ligand-induced downstream signaling (22). Structural studies have further shown that multivalent ligands, such as S100B, enlarge or stabilize RAGE–ligand oligomeric complexes, supporting a model in which receptor clustering initiates signaling (23). Because mRAGE lacks intrinsic kinase activity, oligomerization likely promotes the spatial organization of its cytoplasmic tails and facilitates the recruitment of intracellular effectors, such as DIAPH1, which is required for Rac1/Cdc42-dependent responses (9).

The interaction between the cytoplasmic tail of mRAGE and DIAPH1 couples extracellular ligand recognition to intracellular signal propagation and is required for mRAGE-dependent activation of small GTPases such as RAC1 and CDC42, as well as for downstream cytoskeletal remodeling, ROS generation, and inflammatory responses (9, 24–26). Mechanistically, DIAPH1 acts as a bridge between the mRAGE cytoplasmic tail and the actin-regulatory machinery. Following ligand-induced mRAGE clustering, DIAPH1 recruitment facilitates the activation of Rac1 and Cdc42, leading to actin polymerization, membrane protrusion formation, lamellipodia/filopodia extension, and cell polarization (9). These cytoskeletal changes provide the structural basis for mRAGE-dependent cellular migration, whereas parallel activation of MAPK, AKT, and NF-κB pathways promotes inflammatory and stress-response gene expression. These redox-dependent signals activate MAP kinases, TAK1, the IKK complex, and NF-κB, thereby inducing cytokines, chemokines, adhesion molecules, and RAGE itself (9, 11, 24–26). Accordingly, the mRAGE–DIAPH1 interface represents both a core signaling platform and an attractive pharmacological target, as supported by recent studies of small-molecule antagonists that suppress RAGE-driven inflammation and disease-associated phenotypes *in vivo* (27–29).

In contrast to mRAGE, soluble RAGE isoforms do not transduce intracellular signals because they lack the transmembrane and cytoplasmic domains required for signal propagation. Instead, they function as decoy receptors that bind extracellular ligands and thereby limit ligand access to signaling-competent mRAGE (14, 15, 19). Soluble RAGE comprises at least two major forms. One is shedding-derived soluble RAGE, which is generated predominantly by proteolytic cleavage of mRAGE from the cell surface. Indeed, the majority of circulating sRAGE is derived from cleavage of mRAGE, accounting for more than 50% of total sRAGE, and this shedding process is mediated by metalloproteinases including ADAM10 and MMP9 and may be enhanced under inflammatory conditions (13, 30–32). The other major form is endogenous secretory RAGE (esRAGE), which is generated by alternative splicing and secreted directly as a soluble isoform (14, 15). Because esRAGE retains the extracellular domains and, in principle, also contains a protease-sensitive cleavage site on its C-terminal side, additional proteolytic processing would be expected to generate a soluble product structurally identical to shedding-derived sRAGE. Thus, although sRAGE and esRAGE arise through distinct biosynthetic mechanisms, they can converge to the same final soluble receptor structure and function as ligand-sequestering decoy receptors. Importantly, esRAGE is not restricted to the lung, but is widely distributed across human tissues, including neurons, vascular endothelium, mesothelium, pancreatic β cells, macrophages/monocytes, epithelial structures of the digestive and renal systems, bronchioles, and arterial walls, suggesting broader physiological roles than previously appreciated (33).

Soluble RAGE isoforms add further complexity to the RAGE axis. Total sRAGE comprises both cleaved RAGE and the alternatively spliced endogenous secretory isoform esRAGE, both of which lack the transmembrane and cytoplasmic signaling domains and may function as decoy receptors for RAGE ligands. In the ARIC cohort, low sRAGE predicted incident diabetes, coronary heart disease, and all-cause mortality in individuals with preserved kidney function (34). In addition, long-living individuals show increased esRAGE, suggesting a potential role for esRAGE in healthy aging and longevity (35). In contrast, CARDS and ADVANCE reported that higher sRAGE or esRAGE levels were associated with coronary events, renal outcomes, and mortality in patients with type 2 diabetes (36, 37). This apparent discrepancy may be partly attributable to renal dysfunction, since our group reported a positive association between impaired renal function and circulating esRAGE levels in diabetes, suggesting that renal clearance is a major determinant of circulating soluble RAGE concentrations (32). Thus, elevated sRAGE/esRAGE in high-risk diabetic cohorts may reflect impaired renal clearance and vascular/renal injury rather than increased protective decoy activity alone. Therefore, kidney function should be carefully considered when interpreting soluble RAGE isoforms as clinical biomarkers.

In diabetes and related inflammatory states, chronic ligand accumulation and inflammatory stress appear to perturb the balance between mRAGE and its soluble decoy isoforms. Increased expression of mRAGE, together with altered shedding or splicing, may shift this equilibrium toward heightened signaling competence and greater tissue vulnerability (31). Accordingly, the balance between signaling-competent mRAGE and soluble decoy receptors should be regarded as a critical regulatory checkpoint that shapes both the magnitude and persistence of mRAGE-dependent signaling.

Because RAGE recognizes not only AGEs but also inflammatory DAMP ligands as well as selected PAMPs, these pathways integrate metabolic, inflammatory, and innate immune inputs into a common pathogenic program (11, 19). In this way, mRAGE functions as a signaling amplifier that sustains chronic tissue injury in diabetic complications (**Figure 1**).

## 4 Beyond pathogenic signaling: a physiological role of mRAGE in oxytocin transport and neuroendocrine regulation

While the preceding sections have focused on the pathogenic role of mRAGE as a signaling amplifier in diabetes and inflammation, RAGE also appears to possess an important physiological dimension. This distinction is conceptually significant, because it indicates that RAGE is not solely a mediator of chronic tissue injury, but may also participate in homeostatic processes within the central nervous system (38).

**Figure 2:**
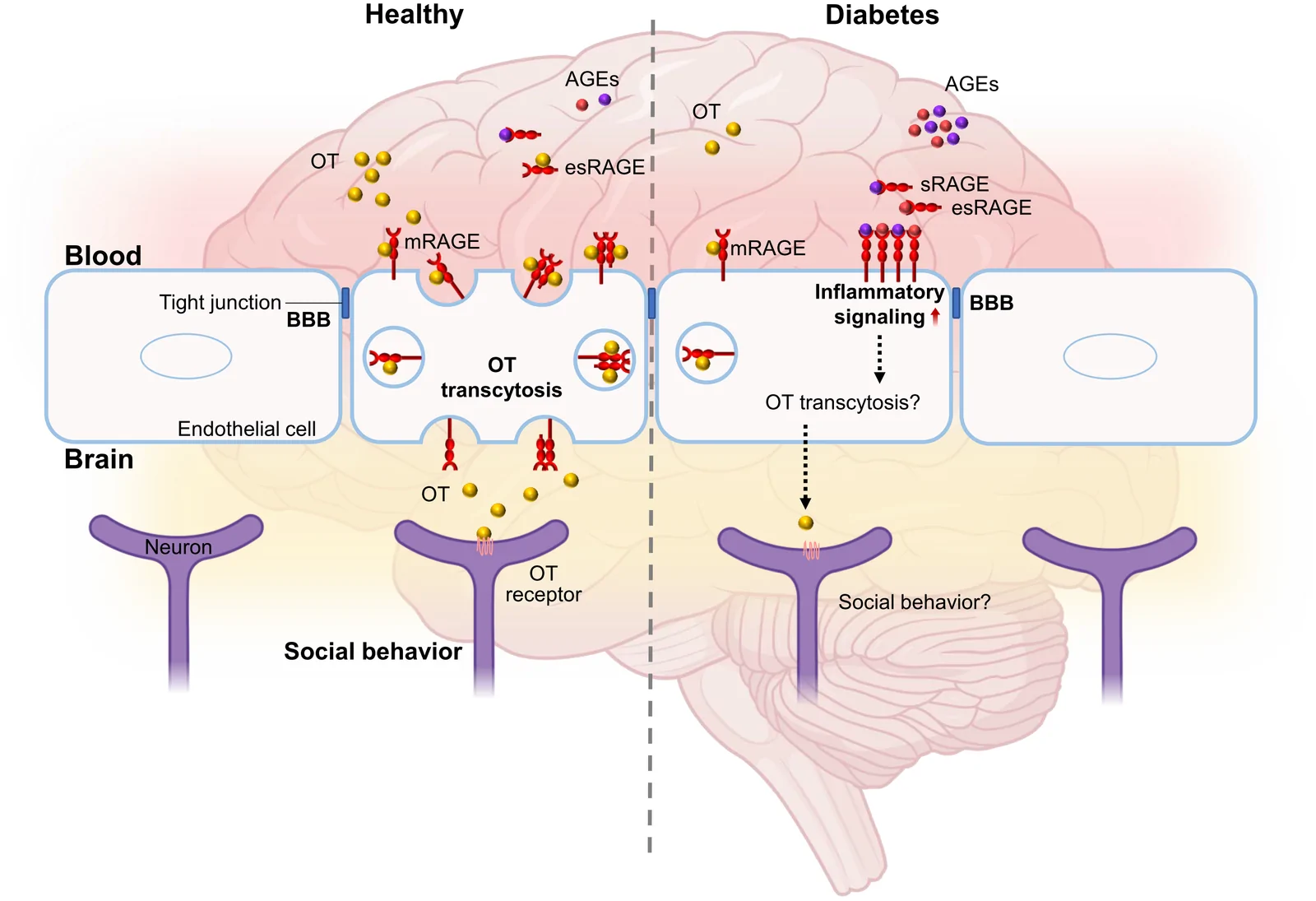
Physiological role of mRAGE in oxytocin transport from the blood to the brain. Membrane-bound full-length RAGE (mRAGE) also mediates the physiological transport of oxytocin (OT) across the blood -brain barrier (BBB). Under healthy conditions, circulating OT is transported from the blood into the brain via endothelial mRAGE. After entering the brain, OT acts on OT receptors expressed in neurons. This pathway contributes to OT-dependent social behavior and neuroendocrine regulation. OT binding to RAGE is mechanistically distinct from the binding of canonical inflammatory RAGE ligands (41). OT does not compete with major pathological RAGE ligands. It does not trigger canonical intracellular RAGE signaling. It also does not interfere with signaling induced by other RAGE ligands. Soluble RAGE isoforms, such as soluble RAGE (sRAGE) and endogenous secretory RAGE (esRAGE), do not appear to function as effective decoys for OT transport (43). Under diabetic conditions, OT production or availability may be reduced, as suggested by clinical and postmortem studies (45–47). In addition, increased AGE accumulation and enhanced inflammatory mRAGE signaling may alter the BBB microenvironment. These changes may affect the efficiency of RAGE-dependent OT transport. Thus, this figure illustrates a potential mechanism by which diabetes may impair central OT signaling through reduced OT availability, altered RAGE-mediated OT transport, and inflammatory neurovascular dysfunction.

One of the clearest examples of this physiological role is the involvement of RAGE in oxytocin (OT) biology. OT is a hypothalamic neuropeptide hormone with established functions in social bonding, emotional regulation, affiliative behavior, and maternal responses (38–40). In addition to its central synthesis and regulated secretion, OT availability in the brain is influenced by transport across the blood–brain barrier (38–40). Notably, mRAGE has been implicated in this physiological transport process, functioning as a carrier for OT from the circulation into the brain (38, 41). Importantly, OT binding to RAGE appears to be mechanistically distinct from the interaction of canonical inflammatory RAGE ligands: it does not compete with other major RAGE ligands, does not trigger canonical intracellular mRAGE signaling, and does not interfere with downstream signaling induced by other RAGE ligands (41, 42). These observations suggest that OT utilizes mRAGE primarily as a transport-associated binding partner rather than as a pro-inflammatory signaling receptor (**Figure 2**) (41, 42).

Recent evidence further supports a role for mRAGE in OT delivery to the brain, including transport to the medial prefrontal cortex after peripheral or intranasal OT administration. At the same time, soluble RAGE isoforms do not appear to function as effective decoys for OT transport (43). In reconstituted blood–brain barrier models, the presence of esRAGE on the luminal side did not inhibit OT transport to the brain compartment, and *in vivo* overexpression of esRAGE did not significantly reduce OT entry into the cerebrospinal fluid (43). These findings suggest that, unlike their inhibitory effects on canonical inflammatory ligands, soluble RAGE isoforms such as sRAGE and esRAGE do not meaningfully interfere with OT transport, at least under the conditions examined.

In addition to mRAGE-dependent transport, complement component 4a (C4a) has recently been identified as an OT-binding protein in plasma, acting as a circulating carrier that modulates free OT availability and may thereby influence brain access of OT and OT-dependent social behavior (44). This finding further suggests that OT dynamics are shaped not only by transport across the blood–brain barrier, but also by interactions with circulating binding partners that regulate the bioavailability of free OT in the bloodstream.

At the same time, this physiological role does not exclude the possibility that pathological mRAGE activation may disrupt neuroendocrine regulation under diabetic conditions. RAGE is expressed in multiple brain regions involved in emotional processing and social cognition, and persistent redox-inflammatory activation may impair neuronal network homeostasis (26). Moreover, oxidative stress, mitochondrial dysfunction, and inflammatory cytokine production within neural and glial cells may adversely affect neuronal excitability, synaptic plasticity, and hypothalamic neuropeptide regulation. Because OT secretion is controlled by intracellular calcium-dependent pathways involving molecules such as CD38 and CD157 (38), chronic metabolic and inflammatory stress may further perturb OT-related signaling. Diabetes has been associated with peripheral and central dysregulation of the OT system. Circulating OT levels are reduced in obesity, newly diagnosed type 2 diabetes, and type 1 diabetes, and are inversely associated with hyperglycemia, insulin resistance, dyslipidemia, and inflammation (45, 46).Consistently, postmortem hypothalamic analyses have revealed a selective reduction of OT-immunoreactive neurons in the paraventricular nucleus of patients with type 2 diabetes, with relative preservation of vasopressin- and CRH-containing neurons (47). Intriguingly, RAGE, beyond its classical role as an AGE receptor mediating diabetic oxidative and inflammatory injury, also transports OT across the BBB from the circulation into the brain. These findings suggest that diabetic OT dysfunction may involve reduced OT availability, selective impairment of hypothalamic OT neurons, and altered RAGE-dependent brain entry of OT (**Figure 2**).

Taken together, these findings suggest a dual perspective on RAGE in neuroendocrine biology. On the one hand, physiological mRAGE supports OT transport and, by extension, OT-dependent social and behavioral functions. On the other hand, chronic ligand-driven mRAGE activation may contribute to neuroinflammatory and redox-mediated disturbances that impair these same circuits. This distinction has important therapeutic implications: strategies aimed at suppressing pathogenic mRAGE signaling should ideally preserve, rather than disrupt, the transport-related physiological role of mRAGE in OT biology. Recognizing this duality extends the AGEs–RAGE framework beyond vascular and metabolic injury alone and highlights the need for context-selective modulation of RAGE function in diabetes and its complications.

## 5 Therapeutic strategies for selective targeting of mRAGE signaling

The preceding sections position mRAGE as a central signaling node that links carbonyl stress, ligand accumulation, oxidative stress, and inflammatory amplification in diabetes. At the same time, the physiological role of RAGE in oxytocin transport suggests that future therapeutic strategies should not indiscriminately suppress all RAGE functions, but instead preferentially target pathogenic mRAGE-dependent signaling. From this perspective, mRAGE signaling represents a particularly attractive therapeutic focus in diabetic complications.

Current therapeutic approaches can be broadly organized into four layers: upstream ligand reduction, extracellular blockade, intracellular interface blockade, and gene silencing.

Upstream ligand reduction aims to limit the generation and accumulation of pathogenic ligands before they engage mRAGE. Approaches in this category include improved metabolic control, suppression of AGEs formation, enhancement of carbonyl detoxification pathways such as glyoxalase activity, and preservation or augmentation of soluble decoy receptor buffering by sRAGE and esRAGE (2–4, 14, 15, 20).

Extracellular blockade focuses on interfering with ligand–mRAGE interactions at the cell surface. Representative compounds include the prototypic antagonist FPS-ZM1 (48) and Azeliragon (TTP488) (49), which advanced into late-stage clinical development. Azeliragon, an orally available small-molecule RAGE antagonist, has provided important clinical information on systemic RAGE inhibition. In a phase II study in mild-to-moderate Alzheimer's disease, lower-dose azeliragon showed potential clinical benefit; however, the higher-dose arm was discontinued because of safety concerns, including increased confusion, falls, and greater cognitive decline. The subsequent STEADFAST phase III trial in mild Alzheimer's disease failed to meet its co-primary endpoints, with no significant improvement in ADAS-Cog or CDR-SB compared with placebo, leading to discontinuation of the Alzheimer's disease program (50, 51). Later subgroup analyses in patients with Alzheimer's disease and type 2 diabetes or impaired glucose tolerance suggested possible metabolically defined effects, but these findings remain exploratory and have not established azeliragon as an effective Alzheimer's disease therapy. More recently, azeliragon has been repositioned for oncology, particularly glioblastoma, where early-phase studies are testing its combination with radiotherapy and/or temozolomide (NCT05635734). Preliminary dose-finding data suggest feasibility and tolerability; however, clinical efficacy remains unproven, and treatment-related adverse events, including grade 3 maculopapular rash, have been reported (52). Therefore, azeliragon supports the druggability of RAGE, while also highlighting key limitations of RAGE antagonism, including dose-dependent tolerability, disease-context dependency, patient selection, and the need for combination strategies. In addition, drug-repositioning approaches have identified agents such as papaverine (53) and low-molecular-weight heparin (LMWH) (54, 55) as modulators of ligand–RAGE interaction. More recently, candidate antagonists such as SBLR876 and benzo[b]thiophene-2-carboxamide derivatives have expanded the medicinal chemistry landscape of direct mRAGE blockade (56, 57). Although many of these agents remain preclinical, they support the tractability of extracellular targeting.

Intracellular interface blockade targets signal propagation downstream of ligand binding. In particular, disruption of the mRAGE–DIAPH1 interaction is especially attractive because it interferes with a convergent signaling node required for downstream activation across a heterogeneous ligand milieu (7, 24, 27, 29). In this context, RAGE229 and RAGE406R provide proof of principle that pharmacological inhibition of the mRAGE–DIAPH1 interface can suppress inflammatory signaling and improve disease-associated phenotypes in preclinical models (27, 29).

Gene silencing represents a fourth strategy aimed at reducing mRAGE expression itself (58). A notable example is ARO-RAGE, an inhaled RNA interference therapeutic designed to suppress pulmonary RAGE expression (59). Early clinical data showing marked reductions in soluble RAGE in serum and bronchoalveolar lavage fluid provide proof of principle that targeted RAGE knockdown is feasible in humans.

Taken together, these approaches indicate that therapeutic development has moved beyond the general concept of RAGE inhibition toward more selective modulation of pathogenic mRAGE signaling. In this regard, strategies that transiently shift the balance from signaling-competent mRAGE toward soluble decoy forms such as sRAGE may represent an especially attractive direction. A short-lived increase in sRAGE generation could attenuate pathogenic ligand-driven signaling without requiring sustained depletion of mRAGE, and might therefore be more compatible with preservation of context-dependent physiological functions such as oxytocin transport. Thus, approaches that promote controlled mRAGE shedding or otherwise induce a temporary shift toward soluble decoy states may offer a therapeutically advantageous balance between suppression of pathological signaling and preservation of physiological RAGE function. Selective and mechanism-based targeting of mRAGE signaling may therefore represent the most promising therapeutic direction for diabetic complications. This Review also highlights the importance of considering RAGE as a context-dependent regulatory system shaped by isoform balance, receptor oligomerization, and physiological transport functions.

## 6 Conclusions and future perspectives

DM is increasingly recognized as a systemic disorder in which metabolic overload extends far beyond impaired glucose regulation to reshape redox balance, carbonyl flux, and inflammatory signaling across multiple tissues (1). Within this broader pathophysiological landscape, the AGEs–mRAGE axis can be viewed as a central organizing framework through which biochemical stress is converted into sustained cellular dysfunction and tissue injury (6). Rather than acting as an isolated pathway, RAGE integrates diverse metabolic, inflammatory, and innate immune inputs into a coordinated signaling network that contributes to the onset and progression of diabetic complications.

This perspective highlights the importance of viewing diabetic complications as the outcome of interconnected processes rather than isolated downstream events. Carbonyl stress, AGEs heterogeneity, and RAGE isoform balance together influence the intensity, duration, and tissue specificity of signaling, thereby shaping how metabolic stress is translated into chronic pathology. Such a framework may also help explain why tissue injury can persist despite improvement in glycemic control and why broader pathway-level intervention may be required. In particular, the balance between signaling-competent mRAGE and soluble decoy isoforms provides a regulatory layer that may determine whether ligand exposure is translated into sustained inflammatory signaling or buffered by non-signaling receptor forms.

At the same time, several fundamental questions remain unresolved. It is still unclear which AGE species, or combinations of AGEs modifications, are most important in driving pathology *in vivo*, how the balance between mRAGE and soluble isoforms is regulated in different tissues, and how mRAGE signaling intersects with neuroendocrine pathways involved in behavior and social function. In particular, elucidating how metabolic stress interfaces with neuronal and behavioral circuits may expand the conceptual framework of diabetic complications beyond traditional vascular endpoints (**Figure 2**).

From a biomarker perspective, HbA1c remains indispensable for diabetes management because it reflects an early glycation product and provides a robust index of cumulative glycemic exposure (1). By contrast, AGEs comprise a heterogeneous class of later glycation products that may more closely reflect cumulative carbonyl stress, metabolic memory, and the tissue injury processes underlying diabetic complications. In principle, AGEs can be assessed by several approaches, including targeted quantification of defined AGE species in blood or urine by chromatographic and mass spectrometric methods, immunochemical assays, and non-invasive estimation of tissue AGEs burden by skin autofluorescence (60). However, important challenges remain unresolved: which AGE species, or which combination of AGE species, is most informative; whether AGEs-based biomarkers can serve as early indicators of diabetic complications; and which analytical platforms are most suitable for clinical implementation. Clarifying these issues will be essential if AGEs are to be integrated into future biomarker strategies beyond conventional indices such as HbA1c. Similarly, soluble RAGE isoforms may have biomarker potential, but their interpretation requires careful consideration of renal clearance, vascular injury, and disease stage. Thus, patient-level stratification based on AGE accumulation, RAGE isoform profiles, renal function, and tissue-specific risk may be important for translating this pathway into clinical practice.

Looking ahead, the most promising therapeutic approaches will likely combine metabolic control with selective modulation of pathogenic mRAGE signaling. Equally important, future therapies should take into account that RAGE also has context-dependent physiological functions, including its contribution to OT transport. This has important translational implications: because RAGE participates not only in pathological inflammatory signaling but also in physiological OT transport across the BBB, complete or prolonged RAGE blockade may not be an optimal therapeutic strategy. In this setting, strategies that transiently reduce signaling-competent mRAGE while increasing soluble decoy forms may be especially attractive. Such an approach could dampen pathogenic ligand-driven signaling without imposing prolonged suppression of mRAGE itself, and may therefore be less likely to interfere with physiological functions that depend on intact RAGE, including OT transport. More broadly, there is a strong rationale for the development of agents that selectively suppress pathogenic mRAGE-dependent intracellular signaling while sparing the transport-related physiological actions of RAGE. Therapeutic strategies with this degree of functional selectivity would be particularly desirable, as they may provide effective inhibition of chronic inflammatory and metabolic amplification without compromising homeostatic neuroendocrine processes. Accordingly, therapeutic modulation of RAGE isoform balance, signaling specificity, and ligand context may represent an important future direction. By linking RAGE isoform regulation, oligomerization-dependent intracellular signaling, biomarker interpretation, and OT transport, this Review supports a more selective translational strategy: targeting disease-driving RAGE signaling while preserving beneficial physiological RAGE functions. Ultimately, re-framing RAGE not simply as a receptor for AGEs, but as a context-dependent regulator of metabolic, inflammatory, and neuroendocrine crosstalk, may provide a more coherent framework for understanding and eventually treating the diverse complications of diabetes.
